# Retraction: Characterization of the complete mitochondrial genome of an important edible fungus *Auricularia polytricha*

**DOI:** 10.1080/23802359.2020.1798637

**Published:** 2020-08-03

We, the Editor, authors and Publisher of *Mitochondrial DNA Part B: Resources*, have retracted the following article:

Qiang Li, Xin Jin, Zuqin Chen, Chuan Xiong, Ping Li & Wenli Huang (2019) ‘Characterization of the complete mitochondrial genome of an important edible fungus *Auricularia Polytricha*’ 4:1, 1107–1108, DOI https://doi.org/10.1080/23802359.2019.1586494.

This article has been retracted at the request of the authors as, subsequent to publication, it has been identified that the species reported in the article is incorrect. The species should be *Suillus sp.* and not *Auricularia polytricha* as reported. The article has therefore been retracted as the data reported in the article are inaccurate.

We have been informed in our decision-making by our policy on publishing ethics and integrity and the COPE guidelines on retractions.

The retracted article will remain online to maintain the scholarly record, but it will be digitally watermarked on each page as “Retracted”.

